# Association of shift work and shift patterns with thyroid function: A NHANES-based cross-sectional study

**DOI:** 10.1097/MD.0000000000050583

**Published:** 2026-09-04

**Authors:** Zeyu Li, Wenjing Peng, Zhiqiang Liao

**Affiliations:** a Department of Thyroid Surgery, The Second Xiangya Hospital, Central South University, Changsha, Hunan, China; b Department of General Surgery, The Second Xiangya Hospital of Central South University, Changsha, Hunan, China; c Department of Gastroenterology, The Affiliated Nanhua Hospital, Clinical Research Center for Metabolic Associated Fatty Liver Disease in Hunan Province, Hengyang Medical School, University of South China, Hengyang, Hunan, China.

**Keywords:** autoimmune thyroiditis, cross-sectional study, NHANES, shift work, subclinical hypothyroidism, thyroid function

## Abstract

This cross-sectional study examined the association between shift work and thyroid function using data from the National Health and Nutrition Examination Survey 2007 to 2010. Weighted univariate and multivariate regression models were applied, with additional subgroup and interaction analyses performed. A total of 3578 participants were included (mean age 41.17 ± 13.31 years). After adjusting for potential confounders, shift workers exhibited significantly higher levels of thyroid-stimulating hormone (TSH) (β = 0.30; 95% CI: 0.14–0.45), thyroglobulin antibody (TgAb) (β = 11.62; 95% CI: 3.49–19.74), and thyroid peroxidase antibody (TPOAb) (β = 8.74; 95% CI: 1.07–16.40), alongside an increased prevalence of autoimmune thyroiditis (AIT) (OR = 1.69; 95% CI: 1.26–2.26) compared with day workers (all *P* < .05). Analyses stratified by shift type revealed that regular evening shifts were most strongly associated with elevated TSH (β = 0.58), subclinical hypothyroidism (SCH) (OR = 2.63), and AIT (OR = 2.29). Additionally, rotating shifts were specifically linked to higher TgAb levels and AIT risk. These findings indicate that shift work, particularly evening and rotating shift patterns, is associated with adverse thyroid-related outcomes.

## 1. Introduction

Thyroid hormones play a crucial role in the normal development and metabolic regulation of various tissues and organs throughout the body. The hypothalamic-pituitary-thyroid axis modulates the synthesis and secretion of these hormones through precise feedback mechanisms.^[1]^ While thyroxine (T4) and triiodothyronine (T3) are the primary hormones secreted, they predominantly circulate in protein-bound states. Their physiological effects are primarily mediated by the unbound fractions – free triiodothyronine (FT3) and free thyroxine (FT4) – which directly interact with cellular targets.^[2]^ Subclinical hypothyroidism (SCH), a prevalent endocrine disorder, is characterized by elevated serum thyroid-stimulating hormone (TSH) levels despite normal FT4 concentrations.^[3]^ The most frequent underlying etiology of SCH is autoimmune thyroiditis (AIT), which affects approximately 10% of the adult population and is more prevalent among women and the elderly.^[4]^ Furthermore, SCH is associated with a spectrum of metabolic disturbances, including increased cardiovascular risk, and its prevalence increases progressively with advancing age.^[5]^

Shift work has emerged as a prevalent and indispensable occupational format in modern society, driven by the escalating demands of the 24-hour economy. This work pattern encompasses evening, night, and other nonstandard schedules. Approximately one-fifth of the global workforce is estimated to be engaged in such labor, with significant concentrations in critical sectors including healthcare, law enforcement, and manufacturing; notably, this proportion continues to rise.^[6,7]^ However, shift work is associated with numerous health risks. Accumulating evidence indicates that irregular work schedules can disrupt circadian rhythms, subsequently impacting metabolic and cardiovascular health and elevating the risks of diabetes, cardiovascular disease, and various malignancies.^[8–14]^ Furthermore, night shift work is linked to diverse mental health issues and functional impairments, such as sleep disorders, chronic fatigue, and depression,^[15,16]^ not only impairing workers’ health and quality of life but also precipitating a decline in productivity with extensive economic and social repercussions.

Several studies corroborate that shift work may exert a profound influence on thyroid function. Epidemiological studies have demonstrated that TSH levels are generally elevated in shift workers relative to day workers, potentially due to circadian rhythm disruption.^[17,18]^ Particularly in work environments characterized by variable circadian cycles, shift workers display marked fluctuations in TSH levels, which are directly linked to increased susceptibility to thyroid dysfunction.^[19]^ Furthermore, according to a meta-analysis, shift employment can raise TSH and FT4 levels, increasing the risk of SCH.^[20]^ Although one Taiwanese study^[21]^ could not identify a strong connection between shift work and SCH, substantial research supports the impacts of shift work on thyroid function, particularly in workers engaged in irregular or prolonged night shifts.^[22]^

The majority of existing research exploring the relationship between shift work and abnormal thyroid function comprises single-center studies with small sample sizes. Furthermore, a lack of detailed categorization for various shift work patterns often precludes the identification of specific schedules that most profoundly impact thyroid function.

Therefore, the primary objective of this study is to investigate the association between various types of shift work and abnormal thyroid function using data from the National Health and Nutrition Examination Survey (NHANES) 2007 to 2010. The secondary aim is to determine which specific shift work schedules pose the greatest risk for thyroid dysfunction. By addressing these objectives, this study seeks to contribute to the existing evidence base and provide insights that may inform future research and clinical management of thyroid health in shift workers.

## 2. Methods

### 2.1. Data source and participants

**Figure 1.**
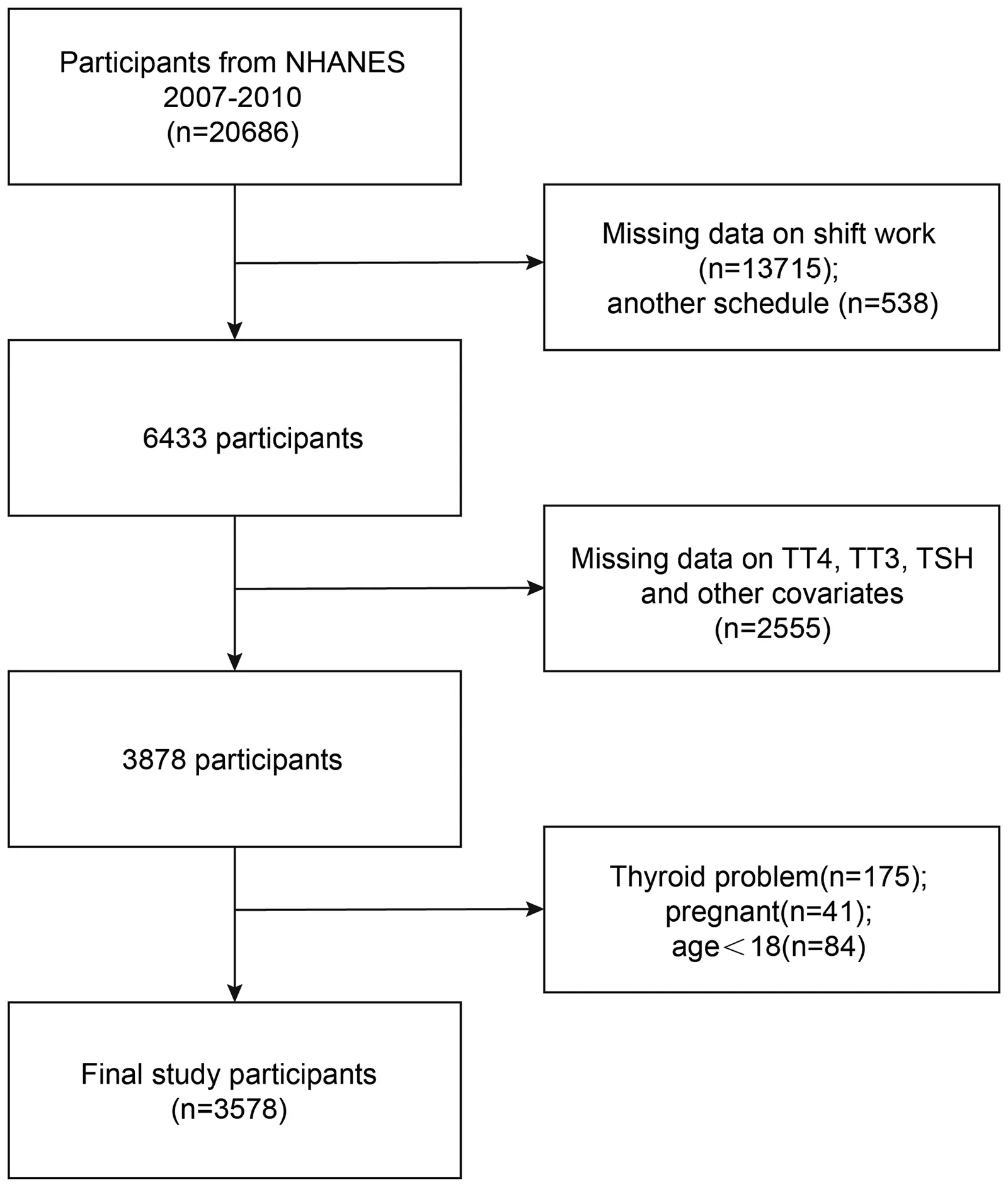
Flowchart of participant selection.

NHANES is a population-based survey in the United States that provides nationally representative data on demographic characteristics, health behaviors, and nutrition, with all protocols approved by the National Center for Health Statistics Research Ethics Review Board. Of the 20,686 participants in the 2007 to 2010 NHANES cohort, 13,715 were excluded due to missing shift-work data. This exclusion primarily stems from the survey design targeting only active workers (excluding retirees and students) and a high item non-response rate among eligible participants. Furthermore, we excluded individuals for another schedule (n = 538), absent thyroid function data (n = 2555), a history of thyroid disease or cancer (n = 175), pregnancy (n = 41), and being under 18 years of age (n = 84), resulting in a final analytical sample of 3578 participants. The sample selection flowchart is depicted in Figure 1.

### 2.2. Laboratory variables

Serum samples were analyzed to assess thyroid-related biomarkers, including FT3, FT4, total T3, total T4, TSH, thyroglobulin antibody (TgAb), and thyroid peroxidase antibody (TPOAb). All thyroid biomarkers were measured following standardized NHANES protocols with rigorous quality control to minimize inter-assay variability. For total T4, which involved a laboratory change during the survey period, statistical harmonization (fractional polynomial regression) was applied to ensure comparability of results across cycles.

### 2.3. Definition of thyroid function

Serum TSH values above the upper reference limit were used to categorize hypothyroidism; normal FT4 levels indicated SCH, whereas low FT4 levels were indicative of overt hypothyroidism. Serum TSH levels below the lower reference range were indicative of hyperthyroidism; normal FT4 levels suggested preclinical hyperthyroidism, while high FT4 levels indicated overt hyperthyroidism. Serum TSH levels within the designated reference range and normal FT4 levels were considered indicators of normal thyroid function. TgAb positivity was determined at levels ≥ 4 IU/mL and TPOAb positivity at levels ≥ 9 IU/mL.^[23,24]^ TPOAb > 9 IU/mL or TgAb ≥ 4 IU/mL were used to define AIT. TSH levels > 4.5 mIU/L and FT4 levels within the standard range of 9 to 25 pmol/L were used to define SCH. TSH values <0.4 mIU/L, FT4 levels between 9 and 25 pmol/L, and FT3 levels between 2.5 and 3.9 pg/mL were indicative of subclinical hyperthyroidism. TSH values >4.5 mIU/L and FT4 levels <9 pmol/L were indicators of overt hypothyroidism. Overt hyperthyroidism was characterized by TSH levels < 0.4 mIU/L, with FT4 levels exceeding 25 pmol/L or FT3 levels surpassing 3.9 pg/mL.^[25,26]^

### 2.4. Shift work type

Information on work schedules was obtained from the Occupational Questionnaire Component of NHANES, which collects detailed data on employment characteristics. Participants were asked to identify their usual working hours at their primary job, with response options including regular daytime, regular evening, regular night, rotating shifts, and other schedules. The “other schedules” category, assigned only after probing to exclude the 4 predefined shift types, was excluded due to its heterogeneous composition and limited sample size (n = 538). This approach is consistent with previous studies.^[27]^ For analytical purposes, individuals working regular evening, regular night, or rotating shifts were categorized as shift workers, while those reporting regular daytime schedules were classified as day workers.^[6,28,29]^

### 2.5. Covariate

The analyses were adjusted for multiple covariates, including age, educational attainment, race/ethnicity, body mass index, family poverty level (FPL), marital status, sex, hypertension, diabetes, smoking, alcohol consumption, urinary iodine concentration categorized as <100 μg/L, 100 to 300 μg/L, ≥300 μg/L, serum cholesterol, weekly work time, and sleep duration. Hypertension was defined as either a medical diagnosis or the use of antihypertensive medication as reported by participants. Diabetes was defined by self-report, antidiabetic treatment, HbA1c ≥ 6.5%, or fasting glucose ≥ 7.0 mmol/L. Alcohol consumption was characterized as heavy drinking, defined as the consumption of 5 or more alcoholic beverages daily over the past 12 months, reflecting recent consumption patterns. A person was considered to be smoking if they had smoked a minimum of 100 cigarettes in their lifetime. The number of working days and the amount of hours worked each day were multiplied to determine the weekly work time.

### 2.6. Statistical analysis

The R software package (version 3.4.3) was used for statistical analyses. MEC exam weights (WTMEC2YR) were used in this study’s analysis due to the intricate multi-stage sample architecture of NHANES. Categorical variables were summarized as weighted proportions, and continuous variables were expressed as weighted means with standard deviations. Group comparisons for baseline characteristics were conducted using the weighted chi-square test for categorical variables and one-way analysis of variance for continuous variables. Before performing one-way analysis of variance, the assumptions of normality and homogeneity of variance were verified using Shapiro–Wilk and Levene tests, respectively. Multivariable logistic regression models were further applied to evaluate the association between shift work and thyroid outcomes. Subgroup analyses and tests for interaction were conducted to assess potential effect modification. To address the potential bias from missing data, we performed multiple imputation by chained equations. A sensitivity analysis was conducted using the pooled results from 5 imputed datasets to ensure the robustness of our primary findings. All analyses were based on two-sided tests, with statistical significance defined as a *P* value < .05.

## 3. Results

### 3.1. Participant characteristics

Data from 3578 participants in NHANES 2007 to 2010 were included in the analysis. Participants were 56.14% male and 43.86% female, with a mean age of 41.17 ± 13.31 years. As indicated in Table 1, shift workers exhibited significantly higher TgAb levels, AIT, and SCH compared to day workers. Age, education level, race, FPL, marital status, hypertension, serum cholesterol, and sleep duration were all shown to differ significantly between the 2 groups. However, there were no appreciable variations between the groups in terms of gender, body mass index, alcohol consumption, smoking, diabetes, urinary iodine concentration, weekly work time, TSH, TPOAb, subclinical hyperthyroidism, overt hypothyroidism, or overt hyperthyroidism. It should be noted that these descriptive comparisons are unadjusted for potential confounders; the associations presented in subsequent sections are derived from multivariable regression models that account for covariates.

**Table 1:** Characteristics of study participants.

Characteristics	Work type		*P*-value
	Day worker	Shift worker	
Age (yr)	42.42 ± 12.89	35.81 ± 13.75	**<.001**
Gender (%)			
Male	56.43	54.88	.47
Female	43.57	45.12	
Race (%)			
Non-Hispanic white	69.2	61.85	**<.001**
Non-Hispanic black	8.51	16.41	
Mexican American	9.82	9.48	
Other races	12.48	12.26	
Education level (%)			
Less than high school	15.74	18.56	**<.001**
High school	23.16	32.66	
More than high school	61.1	48.78	
Marital status			
Married or cohabitating	70.46	53.4	**<.001**
Widowed, divorced, or separated	15.33	33.03	
Never married	14.21	13.57	
FPL (%)			
<1	12.71	24.45	**<.001**
1–3	33.37	36.53	
≥3	53.92	39.02	
BMI (kg/m^2^) (%)			
<25	33.07	34.3	.20
25–30	35.8	32.19	
≥30	31.12	33.51	
Hypertension (%)			
No	77.43	83.67	**<.001**
Yes	22.57	16.33	
Diabetes (%)			
No	95.12	95.38	.77
Yes	4.88	4.62	
Smoking (%)			
No	56.55	52.89	.10
Yes	43.45	47.11	
Alcohol consumption (%)			
No	18.94	20.15	.51
Yes	81.06	79.85	
UIC (μg/L) (%)			
<100	31.79	31.02	.65
100–300	49.38	51.3	
≥300	18.83	17.68	
Autoimmune thyroiditis			
No	88.81	85.35	**.01**
Yes	11.19	14.65	
Overt hypothyroidism			
No	99.08	98.9	.68
Yes	0.92	1.1	
Overt hyperthyroidism			
No	99.77	99.86	.66
Yes	0.23	0.14	
Subclinical hypothyroidism			**.007**
No	97.92	96.15	
Yes	2.08	3.85	
Subclinical hyperthyroidism			.42
No	98.62	99.01	
Yes	1.38	0.99	
TgAb (IU/mL)	6.11 ± 56.48	13.72 ± 122.94	**.02**
TPOAb (IU/mL)	15.58 ± 73.11	20.13 ± 86.07	.16
TSH (mIU/L)	1.83 ± 1.41	1.92 ± 1.76	.18
Serum cholesterol (mg/dL)	196.65 ± 39.43	189.31 ± 39.63	**<.001**
Weekly work time (h)	41.65 ± 12.86	40.77 ± 16.72	.12
Sleep duration (h)	6.83 ± 1.16	6.67 ± 1.42	**.002**

Bold values indicate statistical significance (*P* < .05).

BMI = body mass index, FPL = family poverty level, TgAb = thyroglobulin antibodies, TPOAb = Thyroid peroxidase antibodies, TSH = thyroid stimulating hormone, UIC = urine iodine concentration.

### 3.2. Associations between shift work and TSH, SCH

When comparing shift workers to day workers, there was no discernible relationship between TSH levels and SCH in the unadjusted model. However, upon further categorization of shift types, it was found that regular evening shifts were significantly associated with elevated TSH levels and increased prevalence of SCH compared to day workers, with β = 0.26 (95% CI: 0.05–0.46, *P* = .01) and OR = 2.25 (95% CI: 1.13–4.47, *P* = .02). Notably, after adjusting for potential confounders in Model 3, results indicated that TSH levels were significantly higher in shift workers compared to day workers (β = 0.30; 95% CI: 0.14–0.45; *P* < .001). Upon further categorization of shift workers, it was determined that regular evening shifts were substantially linked to higher TSH (β = 0.58; 95% CI: 0.33–0.82, *P* < .001) and SCH (OR = 2.63; 95% CI: 1.06–6.56, *P* = .04) compared to day workers (Table 2).

**Table 2:** Association between shift work and thyroid stimulating hormone and subclinical hypothyroidism.

	Model 1 β/OR (95% CI)	*P*-value	Model 2 β/OR (95% CI)	*P*-value	Model 3 β/OR (95% CI)	*P*-value
TSH (mIU/L)						
Shift worker						
No	Reference		Reference		Reference	
Yes	0.09 (−0.04–0.21)	.18	0.19 (0.07–0.32)	**.003**	0.30 (0.14–0.45)	**<.001**
Work category						
Day worker	Reference		Reference		Reference	
Regular evening shift	0.26 (0.05–0.46)	**.01**	0.36 (0.16–0.57)	**<.001**	0.58 (0.33–0.82)	**<.001**
Regular night shift	−0.01 (−0.25–0.23)	.95	0.11 (−0.13–0.35)	.35	0.28 (−0.02–0.57)	.07
Rotating shift	0.01 (−0.17–0.19)	.92	0.11 (−0.07–0.29)	.22	0.11 (−0.10–0.32)	.31
Subclinical hypothyroidism						
Shift worker						
No	Reference		Reference		Reference	
Yes	1.31 (0.77–2.22)	.32	1.59 (0.93–2.74)	.09	1.80 (0.95–3.42)	.07
Work category						
Day worker	Reference		Reference		Reference	
Regular evening shift	2.25 (1.13–4.47)	**.02**	2.74 (1.35–5.52)	**.005**	3.03 (1.29–7.12)	**.01**
Regular night shift	0.85 (0.26–2.75)	.79	1.03 (0.32–3.33)	.97	1.61 (0.47–5.49)	.45
Rotating shift	0.92 (0.39–2.15)	.85	1.13 (0.48–2.67)	.78	1.12 (0.39–3.21)	.83

Bold values indicate statistical significance (*P* < .05).

Model 1 was adjusted for none. Model 2 was adjusted for gender, age, and race. Model 3 was adjusted for age, gender, race, education level, marital status, FPL, BMI, hypertension, diabetes, smoking, alcohol consumption, UIC, serum cholesterol, weekly work time, and sleep duration.

BMI = body mass index, CI = confidence interval, FPL = family poverty level, OR = odds ratio, TSH = thyroid stimulating hormone, UIC = urine iodine concentration.

### 3.3. Associations between shift work and TgAb, TPOAb, AIT

In the unadjusted model, shift workers were significantly associated with elevated TgAb levels (β = 7.61; 95% CI: 1.41–13.81; *P* = .02) and an increased prevalence of AIT (OR = 1.29; 95% CI: 1.02–1.64; *P* = .04) compared to day workers. Further categorization of shift workers indicated that regular evening shifts were significantly linked to an elevated prevalence of AIT (OR = 1.62; 95% CI: 1.13–2.33; *P* = .01), while rotating shifts were significantly correlated with higher TgAb levels (β = 14.21; 95% CI: 5.41–23.02; *P* = .002) compared to day workers. In Model 3, compared to day workers, shift workers were significantly associated with higher TgAb (β = 11.62; 95% CI: 3.49–19.74; *P* = .005), elevated TPOAb levels (β = 8.74; 95% CI: 1.07–16.40; *P* = .03), and an increased prevalence of AIT (OR = 1.69; 95% CI: 1.26–2.26; *P* < .001). Further categorization showed that regular evening shifts significantly correlated with a higher prevalence of AIT (OR = 2.29; 95% CI: 1.47–3.57; *P* < .001), and rotating shifts were markedly associated with elevated TgAb levels (β = 18.73; 95% CI: 7.45–30.02; *P* = .001) and an increased incidence of AIT (OR = 1.53; 95% CI: 1.02–2.30; *P* = .04) compared to day workers (Table 3).

**Table 3:** Association between shift work and thyroglobulin antibodies, Thyroid peroxidase antibodies and autoimmune thyroiditis.

	Model 1	*P*-value	Model 2	*P*-value	Model 3	*P*-value
	β/OR (95% CI)		β/OR (95% CI)		β/OR (95% CI)	
TgAb (IU/mL)						
Shift worker						
No	Reference		Reference		Reference	
Yes	7.61 (1.41–13.81)	**.02**	9.08 (2.74–15.43)	**.005**	11.62 (3.49–19.74)	**.005**
Work category						
Day worker	Reference		Reference		Reference	
Regular evening shift	6.32 (−3.81–16.44)	.22	7.53 (−2.69–17.75)	.15	10.54 (−2.69–23.77)	.12
Regular night shift	−3.28 (−15.24–8.67)	.59	−1.03 (−13.06–11.01)	.87	−1.98 (−17.95–13.99)	.81
Rotating shift	14.21 (5.41–23.02)	**.002**	15.46 (6.56–24.36)	**<.001**	18.73 (7.45–30.02)	**.001**
TPOAb (IU/mL)						
Shift worker						
No	Reference		Reference		Reference	
Yes	4.55 (−1.82–10.92)	.16	6.53 (0.05–13.02)	**.05**	8.74 (1.07–16.40)	**.03**
Work category						
Day worker	Reference		Reference		Reference	
Regular evening shift	3.45 (−6.91–13.81)	.51	5.28 (−5.14–15.70)	.32	9.99 (−2.44–22.42)	.12
Regular night shift	0.23 (−12.03–12.49)	.97	3.99 (−8.31–16.28)	.53	8.46 (−6.57–23.48)	.27
Rotating shift	7.66 (−1.45–16.76)	.10	8.81 (−0.34–17.96)	.06	7.97 (−2.74–18.69)	.14
Autoimmune thyroiditis						
Shift worker						
No	Reference		Reference		Reference	
Yes	1.29 (1.02–1.64)	**.04**	1.49 (1.16–1.91)	**.002**	1.69 (1.26–2.26)	**<.001**
Work category						
Day worker	Reference		Reference		Reference	
Regular evening shift	1.62 (1.13–2.33)	**.01**	1.97 (1.35–2.86)	**<.001**	2.29 (1.47–3.57)	**<.001**
Regular night shift	0.95 (0.58–1.55)	.84	1.13 (0.69–1.87)	.62	1.27 (0.70–2.30)	.43
Rotating shift	1.26 (0.90–1.76)	.18	1.39 (0.98–1.96)	.06	1.53 (1.02–2.30)	**.04**

Bold values indicate statistical significance (*P* < .05).

Model 1 was adjusted for none. Model 2 was adjusted for gender, age, and race. Model 3 was adjusted for age, gender, race, education level, marital status, FPL, BMI, hypertension, diabetes, smoking, alcohol consumption, UIC, serum cholesterol, weekly work time, and sleep duration.

BMI = body mass index, CI = confidence interval, FPL = family poverty level, OR = odds ratio, TgAb = thyroglobulin antibodies, TPOAb = Thyroid peroxidase antibodies, UIC = urine iodine concentration.

### 3.4. Subgroup analyses

Subgroup analyses were conducted to assess potential effect modification of the association between shift work and thyroid outcomes (Table 4). No statistically significant interactions were observed between shift work and gender, age quantile, race, FPL, body mass index, or smoking status for AIT. Similarly, no significant interactions were detected between shift work and these subgroup variables for TSH (all *P* for interaction > .05). While the interactions were not statistically significant (*P* for interaction > .05), the association between shift work and AIT remained robust in females (OR = 2.37; 95% CI: 1.60–3.51) but was attenuated in males. Similarly, the impact on TSH levels was primarily observed in the youngest 2 quantiles of age.

**Table 4:** Subgroup analyses of the association between shift work and autoimmune thyroiditis and thyroid stimulating hormone.

Subgroup	AIT OR (95% CI)	*P*	*P* for interaction	TSH β (95% CI)	*P*	*P* for interaction
Gender						
Male	1.11 (0.70–1.74)	.66	.06	0.13 (−0.04–0.30)	.13	.42
Female	2.37 (1.60–3.51)	**<.001**		0.39 (0.06–0.72)	**.02**	
Age quantile						
Q1	2.27 (1.17–4.39)	**.02**	.33	0.58 (0.16–0.99)	**.007**	.07
Q2	2.56 (1.34–4.90)	**.004**		0.22 (−0.11–0.54)	.19	
Q3	1.75 (0.99–3.10)	.05		0.11 (−0.21–0.43)	.50	
Q4	1.11 (0.62–1.98)	.73		−0.07 (−0.43–0.29)	.69	
Race						
Non-Hispanic White	1.47 (0.93–2.32)	.10	.69	0.25 (0.02–0.49)	**.03**	.49
Non-Hispanic Black	2.22 (1.02–4.85)	**.05**		0.06 (−0.15–0.28)	.58	
Mexican American	2.07 (1.14–3.75)	**.02**		0.57 (−0.03–1.17)	.06	
Other races	1.51 (0.75–3.04)	.25		0.15 (−0.28–0.57)	.50	
FPL						
<1	1.78 (1.00–3.17)	.05	.38	0.07 (−0.21–0.36)	.62	.33
1–3	1.32 (0.82–2.14)	.26		0.35 (0.04–0.66)	**.03**	
≥3	2.01 (1.24–3.25)	**.004**		0.27 (−0.01–0.56)	.06	
BMI						
<25	1.94 (1.08–3.47)	**.03**	.58	0.29 (0.01–0.57)	**.04**	.90
25–30	1.45 (0.86–2.44)	.16		0.34 (−0.04–0.72)	.08	
≥30	1.65 (1.05–2.59)	**.03**		0.13 (−0.08–0.35)	0.23	
Smoking						
No	2.00 (1.37–2.93)	**<.001**	.16	0.37 (0.11–0.63)	**.005**	.11
Yes	1.31 (0.83–2.05)	.24		0.07 (−0.15–0.28)	.55	

Bold values indicate statistical significance (*P* < .05).

AIT = autoimmune thyroiditis, BMI = body mass index, CI = confidence interval, FPL = family poverty level, OR = odds ratio, TSH = thyroid stimulating hormone.

### 3.5. Sensitivity analyses

We performed a sensitivity analysis using multiple imputation for missing covariates, with results from the fully adjusted model presented in Table S1, Supplemental Digital Content 1. The estimates were largely consistent with the complete-case analysis: shift work remained significantly associated with higher TSH (β = 0.18; 95% CI: 0.03–0.33; *P* = .02), TgAb (β = 7.08; 95% CI: 0.52–13.63; *P* = .03), TPOAb (β = 8.98; 95% CI: 2.30–15.65; *P* = .008), and AIT (OR = 1.53; 95% CI: 1.19–1.98; *P* < .001). Regular evening shifts were significantly associated with elevated TSH (β = 0.37; 95% CI: 0.13–0.62; *P* = .003), SCH (OR = 3.03; 95% CI: 1.46–6.27; *P* = .003), and AIT (OR = 1.99; 95% CI: 1.36–2.92; *P* < .001), while rotating shifts remained significantly correlated with elevated TgAb (β = 7.31; 95% CI: 0.33–14.29; *P* = .04) and AIT (OR = 1.42; 95% CI: 1.01–2.01; *P* = .05).

## 4. Discussion

This study used data from the NHANES 2007 to 2010 dataset to investigate the relationship between shift work and abnormal thyroid function. After controlling for variables, analysis showed that shift workers had significantly higher levels of TSH, TgAb, TPOAb, and an increased prevalence of AIT than day workers. To address the lack of segmentation in previous research, this study further categorized shift types. Subsequent analysis revealed that, compared to day workers, regular evening shifts were significantly associated with elevated TSH, an increased prevalence of SCH, and AIT. Rotating shifts were also significantly associated with elevated TgAb and AIT.

Studies have connected shift work to thyroid dysfunction, which is characterized by noticeably elevated TSH levels among shift workers compared to non-shift workers, potentially due to disruptions in circadian rhythm.^[19,20]^ Specifically, several studies have identified a significantly increased risk of SCH associated with two-shift and irregular three-shift work schedules.^[22]^ However, certain research, such as a 9-year retrospective cohort study carried out in a Taiwanese hospital, did not find any conclusive evidence linking shift work with SCH.^[21]^ After controlling for pertinent variables, our study identified a positive correlation between shift work and SCH, but this correlation did not reach statistical significance. This variability may be attributed to the diverse types of shift work, including regular evening and night shifts, among others, each potentially leading to different health outcomes. Consequently, we further investigated the relationship between different types of shift work and SCH. Our results demonstrated that regular evening shifts were significantly associated with an increased risk of SCH compared with day schedules, underscoring the potential negative impact on thyroid function. Additionally, our analysis confirmed that shift work was significantly associated with elevated TSH levels, a finding that aligns with the existing literature.

Current research predominantly focuses on the association between shift work, TSH, and SCH, whereas evidence regarding thyroid autoantibodies and AIT remains limited, often based on small sample sizes with inconsistent findings.^[30–32]^ In our study, we observed a strong correlation between shift work and elevated thyroid autoantibodies (TgAb and TPOAb), along with a higher incidence of AIT. These findings indicate that the circadian misalignment inherent in shift work may actively dysregulate the immune system. Indeed, a recent study demonstrated that circadian clock disruption is present in autoimmune thyroiditis and that chronic circadian misalignment can aggravate thyroid inflammatory responses.^[33,34]^ Consequently, the thyroid gland becomes more susceptible to autoimmune attacks, providing a pathophysiological basis for the elevated autoantibodies and increased AIT risk observed in these specific shift patterns.

Our subgroup analysis, while not showing formal interactions, revealed that females and younger individuals might be more vulnerable to the adverse effects of shift work on thyroid function. For female shift workers, this heightened susceptibility may be driven by the complex interplay between sex hormones and immune regulation. Estrogen is known to modulate immune responses,^[35]^ and circadian disruption may exacerbate this interaction, disproportionately amplifying the risk of autoimmune thyroid conditions in women. Furthermore, the more pronounced associations observed in younger age groups could be attributed to a combination of physiological and psychosocial factors. Younger workers often experience higher levels of acute occupational stress as they adapt to new roles, coupled with potentially lower physiological resilience to chronic sleep debt and circadian stressors compared to older, more adapted workers.

The mechanisms underlying the association between shift work and thyroid dysfunction are likely multifactorial, involving a complex interplay of neuroendocrine, stress-related, and behavioral pathways. First, thyroid function is regulated by the hypothalamic-pituitary-thyroid axis,^[1,36]^ which follows a distinct circadian rhythm. Our findings suggest that disruption of this rhythm by shift work may potentially influence hypothalamic-pituitary-thyroid axis stability, as TSH secretion is governed by a central circadian pacemaker that typically peaks at night. Second, beyond circadian misalignment, chronic occupational stress plays a critical role. Shift work often entails high-pressure environments that activate the hypothalamic-pituitary-adrenal axis. This activation leads to elevated cortisol levels, which can subsequently suppress the hypothalamic-pituitary-thyroid axis, thereby blunting TSH secretion or altering peripheral thyroid hormone conversion.^[37–39]^ Furthermore, the association between shift work and thyroid dysfunction may be mediated by behavioral and lifestyle factors. Irregular work schedules are frequently linked to altered eating patterns and dietary choices, which may contribute to metabolic disturbances and insufficient micronutrient intake.^[27,29,40,41]^ These factors, alongside reduced physical activity and increased sedentary behavior, are recognized risk factors that could correlate with abnormal thyroid function.^[42,43]^ These interrelated pathways suggest that the observed associations between shift work and thyroid outcomes are likely multifactorial.

This study has several limitations. First, detailed information on participants’ working conditions, such as shift rotation frequency, rotation periods, and shift intensity, was unavailable, and variability in these factors may have influenced the observed associations. Second, the present study did not examine specific dietary factors, which may have influenced thyroid function and overall health. Third, blood samples were not collected at standardized times, which precluded precise control for circadian variation and may have contributed to measurement variability in hormone levels. Fourth, the substantial exclusion of participants with missing shift-work data may introduce selection bias. Fifth, work schedules were self-reported, which may be subject to recall bias or misclassification. Moreover, this study’s cross-sectional methodology makes it impossible to draw conclusions about causality. Despite adjustment for multiple confounders, residual confounding due to unmeasured factors may persist. Lastly, the outcomes of this study may not be as broadly applicable as they could be since they have not yet been externally validated in separate populations or cohorts.

## 5. Conclusion:

Our study reveals a significant association between shift work and thyroid disorders, particularly among evening and rotating shift workers. These findings suggest the potential value of incorporating thyroid function monitoring into the health surveillance of shift workers. While the cross-sectional nature of this study precludes causal inference, our results provide a preliminary basis for discussing how lifestyle adjustments and work-schedule optimization might potentially support the thyroid health of at-risk populations. Future longitudinal research is essential to validate these associations and to determine the efficacy of targeted intervention strategies.

## Acknowledgments

The contributions of all the participants and all members of study team are greatly acknowledged.

## Author contributions

**Conceptualization:** Zeyu Li, Zhiqiang Liao.

**Data curation:** Zeyu Li, Wenjing Peng.

**Formal analysis:** Zeyu Li, Wenjing Peng.

**Investigation:** Zeyu Li, Wenjing Peng.

**Methodology:** Zeyu Li, Zhiqiang Liao.

**Project administration:** Zhiqiang Liao.

**Resources:** Zhiqiang Liao.

**Software:** Zeyu Li.

**Supervision:** Zhiqiang Liao.

**Writing – original draft:** Zeyu Li.

**Writing – review & editing:** Wenjing Peng, Zhiqiang Liao.


