# Supplementary material for: Association of shift work and shift patterns with thyroid function: A NHANES-based cross-sectional study
Source: Medicine (Baltimore). 2026 Sep 4;105(36):e50583. doi: 10.1097/MD.0000000000050583 (PMC13549512; doi:10.1097/MD.0000000000050583)
Supplement: Supplementary file 1 [file medi-105-e50583-s001.docx]

Table S1. Association between shift work and thyroid function indicators after multiple imputation for missing data.

| **Variables** | **TSH (mIU/L)** |  | **Subclinical hypothyroidism** |  | **TgAb (IU/mL)** |  | **TPOAb (IU/mL)** |  | **Autoimmune Thyroiditis** |  |
| --- | --- | --- | --- | --- | --- | --- | --- | --- | --- | --- |
|  | **β/OR (95% CI)** | ***P*-value** | **β/OR (95% CI)** | ***P*-value** | **β/OR (95% CI)** | ***P*-value** | **β/OR (95% CI)** | ***P*-value** | **β/OR (95% CI)** | ***P*-value** |
| **Shift worker** |  |  |  |  |  |  |  |  |  |  |
| No | Reference |  |  |  | Reference |  |  |  | Reference |  |
| Yes | 0.18 (0.03, 0.33) | **.02** | 1.74 (1.00, 3.03) | .05 | 7.08 (0.52, 13.63) | **.03** | 8.98 (2.30, 15.65) | **.008** | 1.53 (1.19, 1.98) | **<.001** |
| **Work category** |  |  |  |  |  |  |  |  |  |  |
| Day worker | Reference |  |  |  | Reference |  |  |  | Reference |  |
| Regular evening shift | 0.37 (0.13, 0.62) | **.003** | 3.03 (1.46, 6.27) | **.003** | 2.86 (-5.38, 11.10) | .50 | 8.46 (-4.67, 21.59) | .21 | 1.99 (1.36, 2.92) | **<.001** |
| Regular night shift | 0.08 (-0.18, 0.34) | .54 | 1.15 (0.35, 3.80) | .82 | 0.68 (-8.57, 9.93) | .89 | 12.39 (-2.56, 27.33) | .11 | 1.20 (0.72, 1.99) | .49 |
| Rotating shift | 0.10 (-0.10, 0.30) | .34 | 1.24 (0.52, 2.96) | .63 | 7.31 (0.33, 14.29) | **.04** | 9.98 (-1.04, 21.01) | .08 | 1.42 (1.01, 2.01) | **.05** |

All results are based on the fully adjusted model (equivalent to Model 3 in the main text), which adjusted for age, gender, race, education level, marital status, FPL, BMI, hypertension, diabetes, smoking, alcohol consumption, UIC, serum cholesterol, weekly work time, and sleep duration.

Abbreviations: FPL, family poverty level; BMI, body mass index; UIC，urine iodine concentration; TSH, thyroid stimulating hormone.
